# The optic chiasm: a turning point in the evolution of eye/hand coordination

**DOI:** 10.1186/1742-9994-10-41

**Published:** 2013-07-18

**Authors:** Matz Larsson

**Affiliations:** 1 The Cardiology Clinic, Örebro University Hospital, SE − 701 85, Örebro, Sweden; 2 The Institute of Environmental Medicine, Karolinska Institutet, SE-171 77, Stockholm, Sweden

**Keywords:** Evolution, Dexterity, Ipsilateral retinal projection, Multisensory cues, Binocular vision, Optokinetic response, Primate, Predators, Prey

## Abstract

The primate visual system has a uniquely high proportion of ipsilateral retinal projections, retinal ganglial cells that do not cross the midline in the optic chiasm. The general assumption is that this developed due to the selective advantage of accurate depth perception through stereopsis. Here, the hypothesis that the need for accurate eye-forelimb coordination substantially influenced the evolution of the primate visual system is presented. Evolutionary processes may change the direction of retinal ganglial cells. Crossing, or non-crossing, in the optic chiasm determines which hemisphere receives visual feedback in reaching tasks. Each hemisphere receives little tactile and proprioceptive information about the ipsilateral hand. The eye-forelimb hypothesis proposes that abundant ipsilateral retinal projections developed in the primate brain to synthesize, in a single hemisphere, visual, tactile, proprioceptive, and motor information about a given hand, and that this improved eye-hand coordination and optimized the size of the brain. If accurate eye-hand coordination was a major factor in the evolution of stereopsis, stereopsis is likely to be highly developed for activity in the area where the hands most often operate.

The primate visual system is ideally suited for tasks within arm’s length and in the inferior visual field, where most manual activity takes place. Altering of ocular dominance in reaching tasks, reduced cross-modal cuing effects when arms are crossed, response of neurons in the primary motor cortex to viewed actions of a hand, multimodal neuron response to tactile as well as visual events, and extensive use of multimodal sensory information in reaching maneuvers support the premise that benefits of accurate limb control influenced the evolution of the primate visual system. The eye-forelimb hypothesis implies that evolutionary change toward hemidecussation in the optic chiasm provided parsimonious neural pathways in animals developing frontal vision and visually guided forelimbs, and also suggests a new perspective on vision convergence in prey and predatory animals.

## Introduction

**Figure 1:**
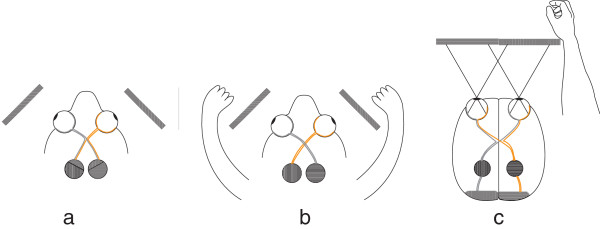
**Schematic of visual pathways in three types of vertebrates: Axonal routing in the optic chiasm is likely to be a reversible process in evolution influenced by the adaptive value of supervising body movements. (a) **Optic pathways in limbless vertebrates*. Snakes and caecilians have developed reduced hemispherical specialization for visually guided steering of the body, and relatively high proportions of ipsilateral retinal projections, similar to phylogenetically less advanced, limbless, vertebrates e.g. cyclostomes [11]. The filled circles represent the superior colliculus (SC). Each hemisphere receives information from both visual hemifields. The SC is a component of the tectum, which integrates visual, somatosensory, and auditory information. It is the mammalian equivalent of the optic tectum in amphibians, reptiles, and birds [3]. **(b) **Optic pathways in a vertebrate with lateralized visual fields and laterally placed forelimbs*. In animals with this anatomy, including the majority of dextropods with limbs, the hemispheres receive practically all, information from the contralateral visual hemifield. The dominance of contralateral retinal projections (CRP) will reduce the need for inter-hemispheric connections, since visual, motor, tactile, and proprioceptive (ViMoTaPro) information concerning the forelimb are processed in the contralateral hemisphere. Thus, when primitive limbless vertebrates began to develop limbs, evolutionary change towards more CRP is likely to have boosted the lateralization of visually guided limbs. **(c) **Optic pathways in a primate*. Due to the architecture of the OC, the hemispheres of primates** receive visual information solely from the contralateral visual hemifield. In species using forelimbs frontally, modification toward ipsilaterality in the temporal retina is associated with corresponding ViMoTaPro areas localized in the same hemisphere. (Only neural pathways to the SC and primary visual cortex are demonstrated). *The rectangles represent portions of the left and right visual hemifield. **Some other animals such as cats, arboreal marsupials, and fruit bats have similar visual systems.

It has been suggested that vision originated as a system for the control of distal movements [1-3]. The primate visual system has a uniquely high proportion (approximately 45%) of retinal ganglial cells that do not cross the midline in the optic chiasm (OC): ipsilateral retinal projections (IRP) (Figure 1c) [4]. The most common explanation is that the proportion of IRP developed in combination with visual convergence due to the selective advantage of accurate depth perception, here called the stereopsis hypothesis [5]. Briefly, the assumption is that binocular viewing creates two slightly different images, due to the positions of the eyes relative to the objects viewed. This binocular disparity provides information that the brain uses to estimate depth in the visual scene [6-9]. An additional idea, the "X-ray" hypothesis, proposed that the degree of binocular convergence maximizes the amount of visual information received, and that primate visual perception is improved by forward facing eyes, since binocularity confers the power of ‘seeing through’ clutter in the visual field [10].

It is commonly suggested that binocular vision and stereopsis in primates was selected for due to the adaptive value of accurate visual control of the hands [5,12]. Harris (1904) [13] stated that “binocular vision is clearly of great assistance in the accurate use of the hand for fine movements….” Others have also proposed an association between binocular vision and eye-hand control [14-16]. According to Goodale [2,3], noteworthy parallels in the functional organization of the subcortical visual system of amphibians, birds, and mammals suggest that independent, closed, and domain-specific processing modules for visuomotor control is an early developing characteristic of vertebrate brains. He proposed the existence of two distinct and interacting systems, vision for perception and vision for action [2,3]. The aim of this review is to explore how stereopsis and other forms of spatial attention are associated with visually mediated motor control of the hands, and to evaluate an eye/forelimb hypothesis in the light of this information.

**Figure 2:**
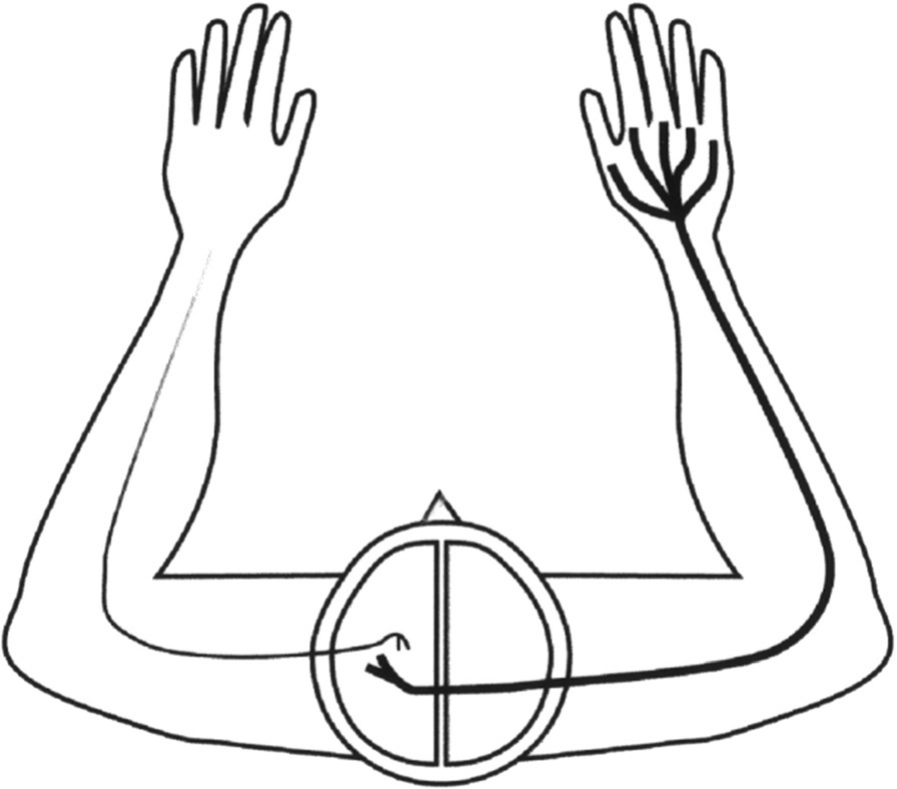
**The motor pathways originating from one hemisphere have a strong contralateral projection that manages both the proximal and the distal musculature. **The ipsilateral projections are not as strong and are involved in only proximal responses [22]. Figure from Gazzaniga M S [22] and used with permission from Oxford University Press.

Vertebrate motor and somato-sensory areas associated with limb movements are largely located in the cerebral hemisphere contralateral to the limb involved (Figure 2). In primates, fibers from the left half of each retina go to the right hemisphere, and the fibers from the right half of each retina go to the left hemisphere. The result is that the left hemisphere receives information from the right visual field, and the right hemisphere receives information from the left visual field (Figure 1c) [17]. Studies have demonstrated that evolutionary processes mediated by regulatory genes may have influenced whether the axons of retinal ganglial cells cross the midline in the OC [18-21]. Retinal projections determine which hemisphere receives visual feedback about an operating forelimb. The EF hypothesis suggests that evolutionary modification of the direction of retinal projections had selective value when the outcome was reduced length of neural pathways of motor and sensory neurons involved in forelimb coordination via the elimination of inter-hemispheric connections in tectal and cortical regions when integrating visual information with somatosensory and motor information about a limb. In addition, it postulates that this principle is a significant mechanism behind the varying proportions of IRP in vertebrate species, and that evolutionary change in the direction of retinal projections may be a reversible process that is influenced by an animal’s need for visual supervision of body movements [12] (Figure 1). The brain hemispheres of limbless species receive a combination of visual information from left and right visual fields (Figure 1a). In species that mainly use the forelimbs in a lateral direction, the visual, motor, tactile, and proprioceptive neurons involved in eye/forelimb coordination are localized in the same hemisphere, with total crossing of retinal ganglial cells in the OC (Figure 1b). Animals such as primates, that have frontal eyes and regularly use the forelimbs in a frontal position also receive visual information in the appropriate hemisphere, but, in this case, it is due to incomplete crossing (hemidecussation) in the OC (Figure 1c) [12]. Since the fundamental architecture of the brains in Figure 1b and 1c is analogous it may be more appropriate to say that short neural pathways among co-working neurons in the primate brain were “preserved” (rather than achieved) through evolutionary change in retinal projections.

Thus, the EF hypothesis proposes that the classic contralateral organization for visually mediated directing of limbs has been preserved in primates through evolutionary change in the visual system. If so, it is likely that components of the visual system that are largely involved in guiding the hand are functionally coupled with other sensory modalities and motor neurons involved in hand coordination. This would predict that the visual system is particularly suited for tasks in the space where the hand usually operates, i.e. the ipsilateral visual field, within arm’s length, and in the inferior portion of the visual field, since reaching movements in primates typically begin in the inferior quadrants of the visual field. This review explores inter-hemispheric communication, the location of the hemidecussation line, the achiasmatic syndrome in humans, IRP influence on oculomotor function, hemispheric specialization in eye-hand control, multimodal perception, and frames of reference in hand coordination. Links between the evolution of stereopsis and the evolution of eye-hand coordination are investigated. Implications for the evolution of vision convergence in mammals are also discussed.

## Review

### Communication between cerebral hemispheres

The primate lifestyle requires frequent relocations in space and coordinated movements of the eyes and hands to interact with objects [23]. Early evidence exists that the anatomy of visual pathways in the OC influences eye-hand coordination. Poffenberger (1912) developed a protocol to investigate sensory-motor integration between hemispheres [24]. Subjects were required to detect lateralized light flashes and to respond by moving either hand. He concluded that when subjects responded with the hand contralateral to the visual stimuli, at least one additional synapse was needed to transfer information from the hemisphere receiving sensory input to that controlling the motor output. The response time difference was presumed to reflect the delay in conduction between the cerebral hemispheres and was designated the crossed/uncrossed difference. Since then, studies have revealed more rapid motor responses to contralateral than to ipsilateral visual stimuli [25-27]. Thus, when for instance the left primate hand operates in the right visual field, the visual directing of the hand must rely on inter-hemispheric connections (Figure 1c). The secondary somatosensory cortex area (S2) appears to be fundamental in interhemispheric information transfer [28,29]. Disbrow et al. [28] suggested that extensive intra-hemispheric processing occurs before information is transferred to the opposite hemisphere.

Ringo et al. [30] proposed that hemispheric specialization developed because the temporal delay in conducting nerve impulses between hemispheres is too great in many instances to permit interhemispheric integration of neuronal computations. Hemispheric specialization means that the neural apparatus necessary to perform each high-resolution, time-critical task is located in a single hemisphere, which results in faster processing and, in addition, optimizes the size of the brain, as the exceedingly large human brain would be even larger without hemispheric specialization [30]. With increasing brain size and greater numbers of neurons, the proportional connectivity decreases, since the number of neurons that each neuron is connected to remains roughly the same [31]. If each neuron in the human brain were connected to every other neuron, its diameter would be some 20 km [32], and metabolic costs would be enormous [33]. Thus, hemispheric specialization has been suggested to be an important principle optimizing the size of the brain while preserving functional connectivity among co-working neurons [30].

### Ipsilateral retinal projections and oculomotor function

**Figure 3:**
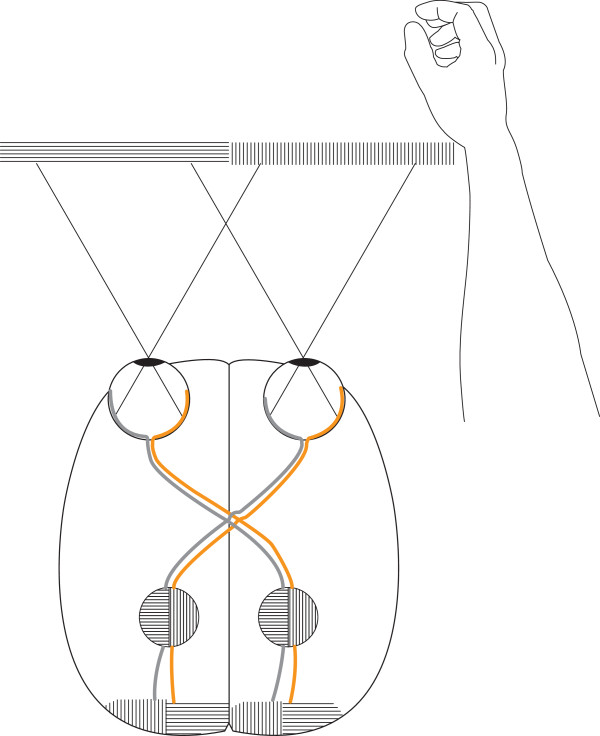
**A primate visual system without ipsilateral retinal projections (IRP). **This hypothesized visual system demonstrates how the primate visual system is likely to function without IRPs. This type of visual system is present in humans with the rare achiasmatic syndrome [36]. In this case the left hemisphere receives a mixture of visual information from the right and left visual field. Binocular cues to the hand’s position in space will be absent in the directing (left) hemisphere, or delayed due to the need for inter-hemispheric information transfer. In a primate without IRP, both eyes and thereby both hemispheres would “see” the hand when it operates in front of the eyes. However, this architecture would be associated with substantial problems for eye-hand steering. In bimanual operations such as climbing, this architecture will provide the left hemisphere with visual information about the right, as well as the left, hand. The latter information would not be particularly useful since it cannot easily be integrated with tactile information, proprioception, motor programming. To make it useful the brain would need more inter-hemispheric connections, which will increase the volume and weight of the brain. This architecture may also have consequences for oculomotor function in monocular conditions, e.g. when a primate gets sudden, transient problems with one eye. If only one eye is used with this neural architecture, visual feedback is likely to be conducted more slowly to the hemisphere that must rely on interhemispheric transfer. The achiasmatic syndrome, as well as albinism, in which the proportion of IRP is significantly reduced [39], are associated with nystagmus [38]. Reduced oculomotor function due to nystagmus is likely to influence eye-hand coordination, which may be fatal in a tree-climbing species.

The achiasmatic syndrome, a rare genetic condition, offers a model of a human vision system without IRP [34,35] (Figure 3). The condition presents clinically with nystagmus [34,35]. In an achiasmatic 15 year old boy, the altered sensory input and mapping was shown to be compensated for to a large extent by interhemispheric communication, although he exhibited disturbance in oculomotor function and lack of stereopsis [36]. In an animal model study of this condition, the preservation of a single binocular representation of the central visual field was shown to be sufficient to prevent the development of nystagmus [37]. In albinism, the decussation line is moved into the temporal retina. This condition is also associated with nystagmus [38].

The optokinetic response refers to eye movement in response to movement in the surroundings, which serves to stabilize the visual image on the retina [40]. Lateral-eyed vertebrates show a characteristic asymmetry of the optokinetic response in the temporal to nasal direction under monocular stimulation, while frontal-eyed vertebrates such as cats and humans exhibit a vigorous optokinetic response in both directions [40]. The mechanism underlying this is unclear. However, a brain without uncrossed fibers in the OC may show asymmetry related problems in transmission of monocular visual information to eye muscle motor nuclei [37]. To produce conjugate eye movements, eye muscle nuclei in the hemispheres must coordinate [41]. Research in goldfish has demonstrated that the brain exploits visual feedback from the environment to fine-tune and stabilize the oculomotor system [42]. Visual feedback is essential in the regulation of saccadic eye-movements in humans [43], and the primate superior colliculus (SC) creates precisely coordinated visual to visuomotor maps related to extra-ocular eye muscle function [44]. When only one eye is used, visual feedback to eye muscle motor nuclei of one hemisphere is likely to be delayed in a primate without IRP, due to the requisite interhemispheric transfer (Figure 3). For example, nerve impulses to both eyes in conjugate eye-movements in right lateral gaze are initiated in the left hemisphere [41]. Due to hemidecussation, both hemispheres will receive visual feedback in a monocular condition. There is evidence that this organization may preserve oculomotor function in case of loss of sight in one eye [32-34].

### The split fovea

**Figure 4:**
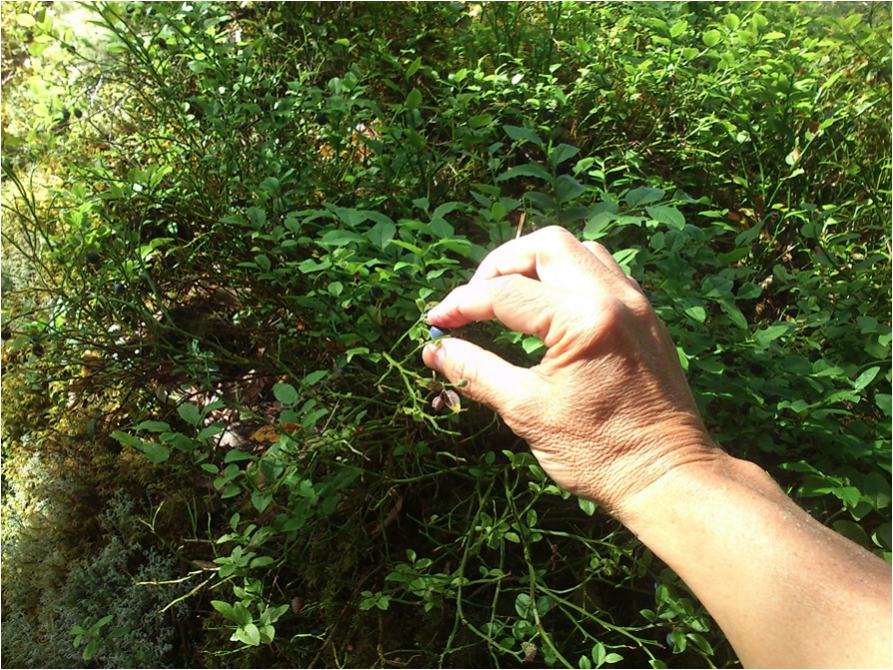
**A primate right hand grip. **A group of neurons that creates a representation of the external world or the own body may resemble a 3 dimensional coordinate system. The object of attention (the berry) might be like *the origin *(the crossing of the x-, y- and z-line). During the reaching maneuver, the right hemisphere receives no primary visual information about the hand. In gripping, the ipsilateral (right) hemisphere receives some visual information (the tip of the thumb and approximately half of the berry). The decussation line primarily follows the thumbnail cuticle. Right hemisphere information about the tip of the thumb, proprioception due to conjugate eye movements and left hemisphere information (touch, proprioception, vision, and motor signals related to the right arm/hand) may create many dimensions in such a coordinate system, contributing to the precision of the hand.

**Figure 5:**
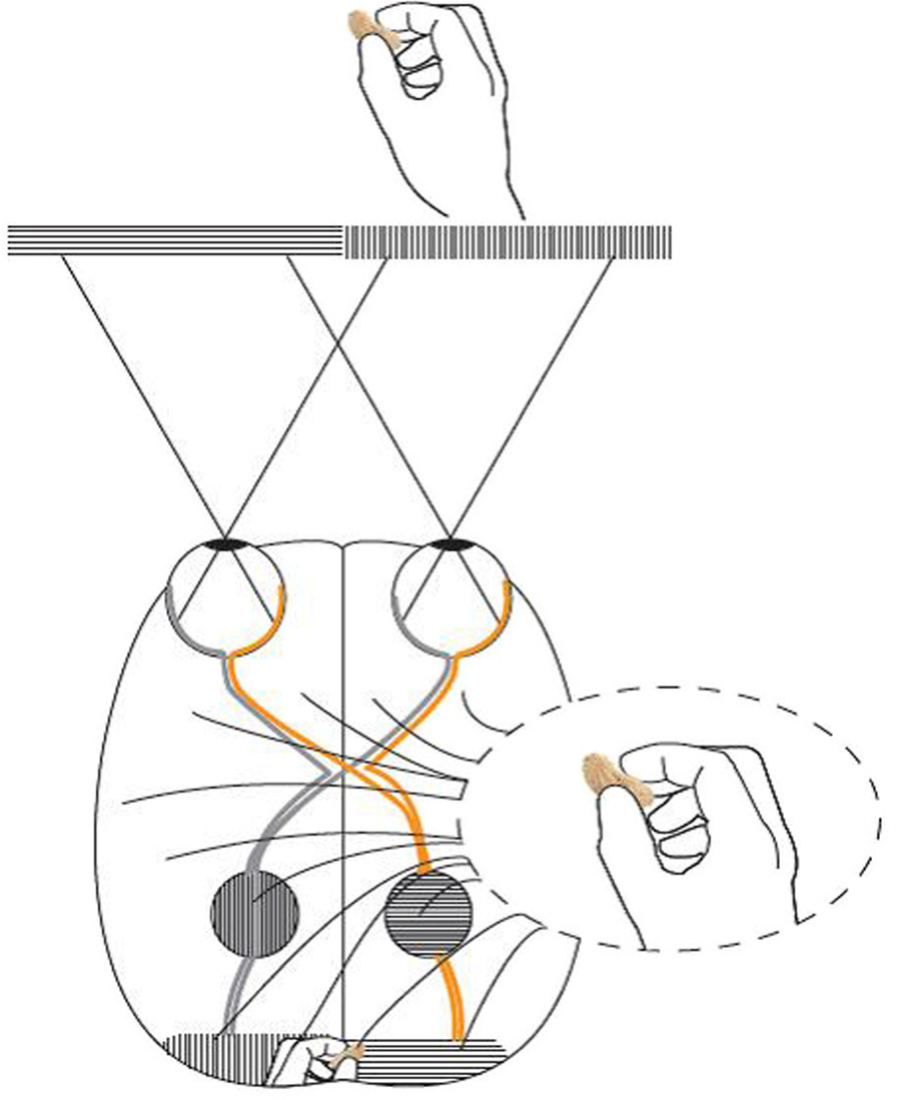
**The split fovea. **A primate focuses on the midpoint of a peanut. The gripping hand is projected in the primary visual area of the hemisphere that steers the hand. The image in the brain schematically demonstrates projections of the hand in the left primary visual area. From this and contributions of other visual areas, the conscious mind creates an image that represents the outer world. Notably, the right hemisphere does not “see” more than the tip of the thumb in this situation, a consequence of the sharp delineation of the visual fields of the left and right hemisphere. An object that is projected approx 0.5° or more to the right of the fovea will not be projected in the left hemisphere [45]. Accordingly, if the eyes shift focus to the wrist, the left hemisphere will receive visual information of the gripping hand only through inter-hemispheric signals.

In humans, the nasal retina projects to the contralateral hemisphere, while the temporal retina projects ipsilaterally (Figure 1c). The line of decussation that divides crossed from uncrossed fibers normally coincides with the vertical meridian through the fovea [38]. Consequently, the right hemisphere receives afferent information from the left visual hemifield, and the left hemisphere receives information from the right visual hemifield. Increasing evidence suggest that the border is sharply delineated, with essentially no overlap of visual information between hemispheres. Harvey [45] presented visual targets to the left and right of vision fixation at various retinal loci and found a difference in reaction time between crossed and uncrossed responses at all loci. Even targets located no more than 0.25° and 0.50° from fixation produced a crossed/uncrossed time difference similar to other loci. This, and results of other studies [46-48], led to the split fovea theory, the assumption that, during fixation, objects presented immediately to the left of fixation are projected to and processed by the right cerebral hemisphere, and objects presented to the right of fixation are projected to and processed by the left cerebral hemisphere [49]. Lavidor and Walsh [49] and Ellis and Brysbaert [50] presented evidence that a split fovea affects reading of words at the fixation point. When fixation falls upon a written word, the letters that fall to the left of the fixation point project initially to the right cerebral hemisphere, whereas the letters that fall to the right of the fixation project to the left hemisphere. The split fovea may have implications for reaching tasks. Under natural conditions, in grasping an object, the eyes fixate on the target before the hand begins to move [51-53]. A consequence is that, when a primate visually fixates on an object before gripping it, visual information of the space between the hand and the object reaches the contralateral hemisphere first. For instance, when the right hand approaches a focused object, only the left hemisphere, which steers the hand, supervises the space through which the hand is moving (Figures 4 and 5).

### Binocular vision in reaching tasks

Binocular cues have since long been considered the most important source of depth information [54-56]. Binocular viewing was shown to result in increased peak reaching velocities [54]. Melmoth and Grant [57] demonstrated that binocular vision is superior to monocular when a human reaches and grips a target. This was shown for real-time execution of the final low-velocity stage of the reach, for coordinating this with target contact, and for all aspects of grasping performance from planning the initial hand posture, through grip closure, to object manipulation. In addition, their results indicated that, when binocular information was unavailable, subjects employed a more cautious strategy, opening the hand wider and more in advance of reaching the target object. Execution of the terminal reach and the grasp remained more inaccurate, error-prone, and variable under monocular than binocular visual control [57].

### Multisensory integration in grasp programming

Keefe *et al*. [58] argued against a special role for binocular vision in grasp programming. They found that grip apertures were smaller when binocular and monocular cues were available than with either cue alone and suggested that this provides strong evidence that the visuo-motor system exploits the redundancy in multiple sources of information and integrates binocular and monocular cues to improve grasping performance. Multisensory integration is fundamental in adaptive behavior since it allows comprehensive perception of the world [59-61]. Visual, auditory, proprioceptive, somatosensory, and vestibular systems influence one another in a complex process from which perceptions emerge as an integrated product [62]. How are objects and events experienced as unitary when they stimulate receptors that give rise to different forms of information? Gibson [63] proposed that dissimilar forms of sensory stimulation are not a problem for the perception of unitary events but rather provide an important basis for it. He argued that the senses should be regarded as a perceptual system with modules working together to access stimulation that is universal across the senses. One type of overlap involves amodal information, which is information that is not specific to a single sense modality but is redundant across more than one sense. For instance the dimensions of time, space, and intensity are typically conveyed by many senses [59], and animal as well as human infants are adept perceivers of amodal information [64]. The anatomy of the OC provides the executing hemisphere with amodal information during visually directed reaching maneuvers. Visual information can be integrated with other modalities further downstream in the same hemisphere. Another consequence is that numerous neurons in the executing hemisphere will fire together, which is commonly proposed to promote learning [65].

### Crossmodal cuing effects in hand coordination

Neurons in the primate SC are active prior to and during arm movements toward visual targets [66]. Neurons in the superficial layers of the SC are responsive nearly exclusively to visual stimuli at specific locations in the contralateral visual hemifield, while the deep layers express sensitivity to sensory stimuli of varying modalities (vision, audition, somatosensation) [67,68]. Multimodal neurons responding to tactile as well as to visual events have been identified in the SC [69-71]. The deep layers of the SC appear to be a coordinating domain [72,73] involved in integration of information and contributing to effective guidance of movements [67,74]. As mentioned, the primate SC has precisely coordinated visual to visuomotor maps related to extra-ocular eye muscle function [44]. Due to IRPs emanating from the lateral retina, motor, tactile, proprioceptive, and visual information of the hand can be integrated in the contralateral region of the SC without interhemispheric communication when the hand operates in the ipsilateral visual hemifield (Figure 1c).

Multimodal neurons responding to tactile as well as to visual events have been observed also in the putamen [75] and in the parietal [76] and premotor [77] cortical regions. Studies utilizing visual images of alien, real, or false limbs have demonstrated that passive viewing of such body-parts can influence the perception of somatosensory stimuli [78,79]. Area 5 in the parietal lobe of the primate brain seems to be involved in monitoring the position and movement of the body. Neurons in this area have been found to encode the position of a monkey's arm while the arm was covered from view. Area 5 neurons responded to the position of a visible realistic false arm, and distinguished a right from a left arm [50,78]. Dushanova et al. [80] observed neurons in the primary motor cortex (M1), an area that is generally considered to initiate and guide movement, that responded to viewed actions. Approximately half of the M1 neurons that were active when monkeys performed a task were also active when they observed a human performing the same action. These so-called ‘view’ neurons were found to be mixed with ‘do’ neurons that are active only during movement [80]. Eisenberg et al. [81] suggested that visual aspects of movement are encoded in M1 only when they are coupled with motor consequences. Notably, when subjects crossed their arms, cross-modal cuing effects were reduced [79,82]. Because visually-based directing of the hands with crossed arms relies on inter-hemispheric communication, crossing arms may simulate a visual system without IRP.

### Neural representations in reaching tasks

It has been suggested that humans and animals form cognitive maps of their environment [83]. Such maps may be sensory or motor, and they may represent the external world or be body representations [74]. An alternative view is the simulation theory (reviewed by Hesslow [84]), which proposes that a simulated action can elicit perceptual activity that resembles the activity that would have occurred if the action had actually been performed. Research has demonstrated a similarity between patterns mapped in the brain and concrete objects [85]. The retro-splenial cortex in humans seems to be directly involved in coordinating and translating egocentric and allocentric frames of reference. The latter is a frame of reference that is centered on a point in space distinct from the space that the perceiver occupies [86-89]. The brain does not create a single unit representation of space, but produces numerous representations of space to achieve stable perception, spatial knowledge, and motor guidance [43]. The process of forming object representations in visual short-term memory from visible characteristics of a stimulus, such as color, shape, size, orientation, location, movement, etc., is referred to as feature binding [90,91]. In contrast to other properties such as color and shape, location plays a key role by providing the spatial map to which individual features are attached, and eventually combined with, to form objects [90,91]. Thus, the location of the object of attention is an important factor in multimodal perception [90,91], and if visual short-term memory is seen as a map or a three dimensional coordinated system, the object of attention seems to have similarities with the origin, i.e. the point where the axes of the system intersect, in a Cartesian coordinate system (Figure 3). Feature-based attention, principally “vision for perception” [2,3], appears to operate across hemispheres [92,93], whereas spatial attention, largely associated with action, appears to operate over local groups of neurons within a hemisphere [92,93].

### Frames of reference in movement planning

Humans are able to grip objects whether the objects are heard, seen, or touched. Consequently, information about the location of objects is recoded in a joint-centered frame of reference, despite of the sensory modality involved [94]. The location of reaching targets may be encoded in an eye-centered frame of reference whether the targets are visual, auditory, proprioceptive, or imaginary [94]. The recalled eye-centered location is updated following each eye and head movement and also when vision is not used, which may reflect a predominant role of vision in human spatial perception [94,95]. Behavioral studies in humans and studies of reach-related cerebral areas in primates have highlighted the dominance of eye-centered coordinates in movement planning [96]. Recent research has revealed that the frame of reference may shift. Parietal area V6A contains neurons modulated by the direction of gaze as well as neurons that code the direction of arm movement. The majority of V6A reaching neurons use a system that encompasses both of these reference frames [97]. The authors suggested that their results “are in line with the view of a progressive visuomotor transformation in the dorsal visual stream that changes the frame of reference from the retinocentric one, typically used by the visual system, to the arm-centred one, typically used by the motor system” [97]. The dorsal aspect of the premotor cortex (PMd) is another area highly involved in visually guided reaching. In the PMd, some neurons encode reaching goals using limb-centered reference frames, others employ eye-centered reference frames, while some cells encode reaching goals in a reference frame by the combined position of the eyes and hand [98]. Mulette et al. reported that, in the intraparietal cortex, the reference frames of individual neurons ranged from predominantly eye-centered to predominantly head-centered, with most neurons reflecting an intermediate, or hybrid, reference frame involving a combination of head- and eye-centered information [99].

Studies of the SC [100], the ventral premotor cortex [101], and the dorsal premotor cortex [102] have identified populations of neurons associated with arm movement that are either clearly eye-centered or consistent with eye-centered coding [96]. Studies of reaching movements to memorized targets in three-dimensional space with visual feedback of the moving extremity suggest a coordinated system that is centered on the line of sight [103-105]. When visual feedback of the hand is altered, subjects alter the arm’s course so that the pathway appears visually straight [106,107]. Functional magnetic resonance imaging studies showed that the human premotor and posterior parietal cortex (PPC) contain neurons that specifically encode visual stimuli close to the hand suggesting that the premotor and PPC are involved in a mechanism for the selective representation of visual stimuli near the body in hand-centered coordinates [108]. In the PPC there is considerable overlap among the regions that are important for spatial working memory, visually guided actions, and navigation, and the PPC contains specialized subunits for the processing of spatial goals of saccades and reaching movements. Together these subunits are commonly labeled the parietal reach-region (PRR), which corresponds primarily to the medial intraparietal cortex [43]. Bhattacharyya et al. [109] showed that, when neurons in the PRR code depth in relation to a fixation point, gaze-centered coordinates are used. Sorrento and Henriques [110] studied the effects of gaze alterations on repeated arm movements toward a fixed target and found that, when the second movement was produced, it was guided by an updated, eye-centered, frame of reference. Based on this and other studies [111,112], Medendorp [43] suggested that gaze-centered coordinates are vital for achieving spatial reliability in the motor system. Hand movements are characteristically initiated before the end of the orienting saccade to a target [113]. This indicates that the peripheral vision information available to plan eye and hand movements relative to a target is the same, and that this information is stored in the visual short-term memory. Thus, the central nervous system may use a common spatial representation of targets to plan both eye and hand movements [114]. Sighting dominance, i.e. the eye that is consistently favored under monocular viewing, has traditionally been considered to be a robust individual trait [115]. However, Khan and Crawford [116,117] found that subjects altered ocular dominance as a function of horizontal gaze direction in a reaching task. Notably, the alternating of ocular dominance depends on which hand is used to reach out and grasp the target [115,117].

### The eye in service of the hand

The EF hypothesis implies that the visual system is well equipped to serve the hand in a reaching task. Vision profoundly influences arm movements soon after birth [118]. The preceding section demonstrated that gaze-centered coordinates are commonly used and essential in the visual directing of the hand [43,94-96,109], and the alternating of ocular dominance in reaching may be another example [116,117]. Reaching movements in primates typically begin in the inferior quadrants of the visual field because of the lower position of the upper limb relative to the visual axis. A bias in spatial discrimination during reaching movements in favor of the lower visual field has been described [119-122]. It was proposed that this may account for the faster manual reaction times reported for the lower visual field; the lower field bias influences the capacity of primates to reach for, grasp, and manipulate objects [123]. Thura et al. [124] demonstrated that hand position influenced saccadic activity in the monkey brain frontal eye field (FEF). Single neurons were recorded in the FEF of two monkeys as they executed a visually guided saccade task while keeping the hand at specific locations on a touch screen. They concluded that visual and proprioceptive signals derived from the hand are integrated by FEF neurons, and showed that hand-related modulation is more pronounced in the lower than in the upper visual hemifield [124]. The medial posterior parietal cortex area, V6A, is proposed to be the earliest node of the dorsal visual stream where visual, eye, and arm position-related information converge [23,115-127]. V6A contains arm movement related neurons that encode the direction of reach [128], hand orientation [129], and grip formation [130]. Hence, multisensory encoding of space is likely to be realized in V6A [23,126]. A predominance of visual neurons with receptive fields typically representing the lower visual field, where the hand usually operates, has been demonstrated in area V6A [131,132]. The conjunction of visual and somatosensory information is considered to form the representation of peripersonal space in many primate brain areas [79,133,134]. Hadjidimitrakis et al. [23] studied neural signals related to binocular eye position in a task requiring monkeys to perform saccades and fixate on targets at various locations in peripersonal and extrapersonal space. They found that neurons in area V6A are sensitive to visual fixation and the location of the foveated target in three-dimensional space, and that they are more highly activated by gaze positions in the peripersonal space. The influence of a vergence signal on fixation has also been reported in the primary visual cortex [135] and in area V4 [136], and in both cases neurons were more activated by fixation points in space within arm‘s length. Viguer et al. suggested that vergent eye movements occur most frequently in the space corresponding to arm‘s length [137].

### The explanatory potential of the EF hypothesis

The concept that binocular vision and abundant IRP result in stereopsis is well established. Studies have used the presence of binocular vision as a verification of stereopsis in, for example, Macropodidae [138] and tyrannosaurs [139]. The wallaby, a small species of the Macropodidae, shows partial decussation of optic nerve fibers and has a binocular field of 50° [138]. This could also serve as evidence for the EF hypothesis, since visual control of forelimbs seems to be common in their foraging behavior [140]. Based on the fossil record, Stevens [139] concluded that Tyrannosaurus rex and other coelurosaurs possessed functional stereopsis. It may be that bipedal coelurosaurs commonly used the forelimbs in the binocular field. The anatomy of Tyrannosaurus rex and Troodon indicates considerable binocular vision below the axis of the head [139], where the forelimbs were likely to operate.

It has been proposed that mammals and birds may use binocular visual fields differently [141,142]. Martin proposed that binocularity in birds does not result in stereopsis, with the possible exception of owls, rather its primary role is control of bill or feet position in foraging [142]. Such visual control of body appendages provides functional analogies with the EF hypothesis [12].

Evidence demonstrates that communication among visual and motor neurons is slower when interhemispheric communication is required [24-27]. Multimodal sensory information used in hand coordination is likely to be transmitted slower in a primate brain without IRP. Moreover, data on multimodal sensory information and a primary role for gaze-centered coordinates in reaching tasks [43,94-96,109] indicate that supervision of the hands is largely integrated with motor control. Neurons in the primary motor cortex responding to viewed actions of a hand [80], visual feedback resulting in modification of arm movements [106,107], multimodal neurons responding to tactile as well as visual events [69-71,75-77], and the reduction of cross-modal cuing effects when arms are crossed [79,82] (simulating a visual system without IRP) also support the EF hypothesis. The primate visual system is highly suited to supervision of tasks where the hand typically operates, within arm‘s length [23,135-137] and in the inferior visual field [119-124,131,132].

The differing proportions of IRP in the non-image-forming visual pathways involved in circadian rhythm and pupillary light responses relative to image-forming pathways [143] can also be accommodated by the EF hypothesis, since non-image-forming pathways do not influence eye/hand coordination [12]. Ipsilateral retinal projections originating only from the temporal retina conform to the hypothesis, since IRP from the nasal retina would increase the need for interhemispheric communication [12]. The EF hypothesis can provide keys to the evolution of IRP in many non-mammalian vertebrates such as the high proportions of IRP in limbless but phylogenetically diverse animal groups such as snakes, caecilians, and cyclostomes (Figure 1a) [12]. The low proportion of IRP in most fishes, birds, and reptiles is in accordance with the EF hypothesis [12]. The premise of depth perception through binocular disparity is largely restricted to mammals [11,12]. The X-ray hypothesis of Changizi and Shimojo [10] does not take into account that early primates were small compared to environmental objects such as leaves, and therefore early primates most likely did not achieve the suggested selective advantage of seeing through environmental objects [5].

### The placement of eyes in primates, predators, and prey

It is commonly suggested that binocular vision is especially useful to predators for estimating the distance to prey, while animals preyed upon often have laterally situated eyes, which provides an ability to scan a broad area of the surroundings without moving the head [144]. The law of Newton-Müller-Gudden (NGM) proposes that the number of optic nerve fibers that do not cross the midline is proportional to the size of the binocular visual field [145]. The majority of predatory mammals have frontally placed eyes and also a significant proportion of IRP [146], while mice, for example, have laterally situated eyes and no more than 3% IRP [18-21]. However, the NGM law has some inconsistencies. Predatory mammals such as dolphins display no IRP [147], and the variation in IRP among non-mammalian vertebrates has been suggested to be inexplicable and to lack association with a predatory lifestyle [11]. Therefore, the EF hypothesis seems to apply to a wider range of organisms than does the NGM law.

Heightened depth perception within the working space of the hand has adaptive value in arboreal primates. Arboreal marsupials, as well as fruit bats that use claws on the wing to manipulate fruits [148-150], possess a primate-like visual system with a high proportion of IRP. The domestic cat’s high proportions of IRP, around 30% [21] vs. 22% in the domestic dog [151], is in accordance with the EF hypothesis, since cats are tree-climbers and extensively direct the forelimbs using vision during prey capture [15].

### Limitations and testability of the hypothesis

The EF hypothesis might be evaluated through comparative analyses of mammalian and non-mammalian associations among IRP, eye convergence, and visual guidance of forelimbs. Ultimately it is DNA that determines whether the axon of a retinal ganglion cell crosses or not [18-21], and transcription factors play vital roles in this process [152-154]. There are indications that visual guidance of forelimbs may have influenced the morphogenesis of the retina and the regionalization of the OC area in numerous vertebrate species [12]. Many molecules and mechanisms involved in OC formation have been conserved in evolution [21], and the EF hypothesis may provide the opportunity to explore associations between visual guidance of forelimbs and alterations in the DNA. A predict of the EF hypothesis is that binocular vision should be expected in animals with forelimbs or similar appendages that habitually operate in front of the animal. This seems to be the case in praying mantises, insects that capture and manipulate prey with powerful forelimbs. The eyes of mantises offer a wide binocular field, and, at close range, precise stereoscopic vision [155]. The proportion of IRP in mantises seems not to have been investigated, and offers opportunity for assessing the EF hypothesis in another phylum. *Octopus vulgaris* may be another candidate. This species has been reported to combine arm location information with visual input to control complex goal directed movements [156].

## Conclusions

This review supports the principle that evolutionary modifications in the proportions of IRP in the primate brain contributed to visual guidance of the hands, and emphasizes that stereopsis is largely associated with visual directing of the hand. Accurate movement of primate forelimbs depends on continuous and reciprocal interaction between motor and sensory systems. Goodale proposed that vision originally developed to control movement [1-3]. This review suggests that visual control of limbs continued to influence the evolution of vertebrate visual systems, and that the combination of convergent vision and increased proportions of IRP was a fundamental factor in the evolution of eye/hand coordination in primates. The EF hypothesis provides a rationale for the localization of eyes in primates and predatory mammals and is applicable in non-mammalian species. In addition, the EF-hypothesis suggests how the classic vertebrate cross-lateralized organization for visually guided limb movements may have been preserved in early primates when they gradually changed their ecological niche to an arboreal lifestyle. It postulates that evolutionary change towards hemidecussation in the OC provided parsimonious and efficient neural pathways in animals with an increasing degree of frontal vision and frontally-directed, visually guided, motor behavior. Further studies may clarify the extent to which the optic chiasm was a turning point in the evolution of stereopsis.

## Abbreviations

OC: Optic chiasm; IRP: Ipsilateral retinal projections; EF hypothesis: Eye-forelimb hypothesis; S2: Secondary somatosensory cortex area; SC: Superior colliculus; M1: Primary motor cortex; PMd: Dorsal aspect of the premotor cortex; PPC: Posterior parietal cortex; PRR: Parietal reach-region; FEF: Frontal eye field; NGM law: Law of Newton-Müller-Gudden.

## Competing interests

The author declares that there are no competing interests.
